# Effects of the kinematic variable, time delay and data length on test–retest reliability of the maximal Lyapunov exponent of human walking

**DOI:** 10.1098/rsos.240333

**Published:** 2024-10-09

**Authors:** Ilseung Park, Jun Hyuk Lee, Jooeun Ahn

**Affiliations:** ^1^ Department of Physical Education, Seoul National University, Seoul, South Korea; ^2^ Institue of Sport Science, Seoul, South Korea; ^3^ Soft Robotics Research Center, Seoul, South Korea

**Keywords:** maximal Lyapunov exponent, test–retest reliability, walking, kinematic data, data length, time delay

## Abstract

The maximal Lyapunov exponent (MLE) has been used to quantify the dynamic stability of human locomotion. The method for estimating MLE requires selecting a proper time series of kinematic variables and reconstructing phase space using proper time delay. The data length also affects the reliability of the measured MLE. However, there has been no criterion for the choice of the time series, time delay or data length. Here, we quantified the effect of these factors on the test–retest reliability of MLE estimations. We recruited 15 young and healthy adults and let them walk on a treadmill three times. We calculated MLE employing various lengths of time series of 18 frequently used kinematic variables and two typical choices of time delay: fixed delay and delay selected by average mutual information algorithm. Then, we measured the intraclass correlation coefficient (ICC) of the measured MLE under each condition. Our results show that the choice of time delay does not affect reliability. Five among the 18 kinematic variables enabled excellent reliability with ICC above 0.9 within 450 strides and also enabled ICC above 0.75 even with 60 or less strides. These findings can contribute to establishing the criteria for measuring the dynamic stability of human walking.

## 1. Introduction

Multiple techniques in applied mathematics have been used to quantify the dynamic properties of human motor behaviours. One flagship example is the use of the maximal Lyapunov exponent (MLE), which measures the dependence on initial conditions of various systems, in evaluating the dynamic stability of human locomotion. Previous studies illustrated that MLE can be used as an early indicator of fall [1–3]. It has also been reported that MLE can differentiate the young from the elderly who are more likely to be exposed to the risk of fall [4,5].

Estimations of MLE from experimental data start with phase space reconstruction, which is the process of constructing a high-dimensional vector using a one-dimensional time series [6]. The phase space is typically reconstructed by using the method of delays as shown in equation (1.1):


(1.1) S(t)=[z(t),z(t+τ),z(t+2τ),...,z(t+(dE−1)τ)],

where *S*(*t*) is the reconstructed vector, *z* is the selected one-dimensional time series, *τ* is the time delay, and *dE* is the embedding dimension.

Theoretically, MLE should be invariant with respect to time delay under ideal conditions [7]. However, unlike determined systems such as the Lorenz attractor, human gait data inevitably contain inherent noise. Additionally, the data of human locomotion have limited lengths because it is often difficult for humans to walk for sufficiently long periods; this factor makes the estimation of MLE from human walking data more challenging compared to the estimation of MLE from other data such as fluid dynamics and economics. The presence of noise and the limited data length often distort the value of MLE, even with consistent phase space reconstruction [8]. Consequently, methods for reconstructing state space with human gait data have been refined over the years to address these challenges.

However, there has been neither agreement nor guidance on how to construct the phase space. In particular, the choices of the kinematic variable that forms the one-dimensional time series, the length of time series and the time delay differ across various studies. Some previous studies differentiated between the gait-impaired and healthy groups using the time series of the trunk acceleration [1,3,9–12], whereas other studies used the time series of the trunk velocity [2,4]. Likewise, multiple studies suggested the minimum length of time series as 150 strides or the length from 2 to 3 min walking [13,14], whereas other studies differentiated fall-prone groups from the healthy with no more than 30 strides [1,5]. For the time delay, several studies employed the average mutual information (AMI) algorithm [1,3,10], whereas various other studies employed a fixed delay without using the AMI algorithm [2,4,9,11,15].

Despite these disagreements, the combined effects of the chosen kinematic variable, data length and time delay on the reliability of the MLE estimations have not been studied systematically. Although several studies addressed the individual effects of the type of kinematic variables [1,3,4,10,16], data length [13,17,18] and delay [19], the possible interaction effects owing to the combination of the choices of the three factors have not been quantitatively assessed yet. This lack of the quantification of the combined effects of the three factors on MLE estimations hinders the development of any convincing guideline on the experimental set-up for MLE estimations of human walking.

In this study, we aim to investigate how the choices of the kinematic variable, data length and time delay affect the reliability of MLE estimations. Fifteen young and healthy participants walked more than 450 strides on a treadmill three times. We estimated MLE of their walking by employing widely used time series of trunk velocity and acceleration, various data lengths between 10 and 450 strides and the time delay extracted from two common methods (the AMI algorithm and fixing the delay as 10% of the gait cycle) [20]. Then, we assessed the test–retest reliability of MLE estimations for all possible combinations of the employed time series, data length and time delay.

## 2. Methods

### 2.1. Participants

We recruited 15 young and healthy male participants (age of 24.4 ± 2.0 years, body mass of 77.0 ± 7.2 kg and height of 1.78 ± 0.06 m). None of the participants reported neuromuscular or biomechanical issues in the lower extremities. The study was conducted in accordance with the laws and ethical standards established by the Declaration of Helsinki, and the Institutional Review Board of Seoul National University approved the study protocol (IRB no. 2205/002-009). Participants were informed about the procedure, and they provided written consent before participating in the experiment.

To determine an appropriate sample size, we conducted a power analysis using the ICC.Sample.Size package in R, based on the methodology by Zou [21]. We set the expected intraclass correlation coefficient (ICC) (*p*) to 0.9 and the null hypothesis ICC (*p0*) to 0.7, with three raters (*k*): a significance level (alpha) of 0.05, one-tailed test and desired power of 0.80. The calculation indicated that a sample size of 13 subjects is required to achieve the desired power. To ensure sufficient statistical power, we decided to recruit 15 participants.

### 2.2. Experimental procedure

Before conducting the experiment, the preferred walking speed (PWS) of each participant was estimated as follows. First, we set the treadmill speed as 0.5 m s^−1^ and let the participants walk for 20 s. We gradually raised the treadmill speed in a stepwise manner with intervals of 0.05 m s^−1^ until the participants reported that the speed reached their usual walking speed. After we recorded the speed, we raised the treadmill speed to 1.6 m s^−1^, which is 140% of the average PWS reported in a previous study [22] and let the participants walk for 20 s. Then, we lowered the speed until the participants reported that the speed reached their usual walking speed. This protocol was repeated three times. The PWS was calculated as the average of the six recorded speeds which the participants indicated as their PWS during the protocol.

**Figure 1.**
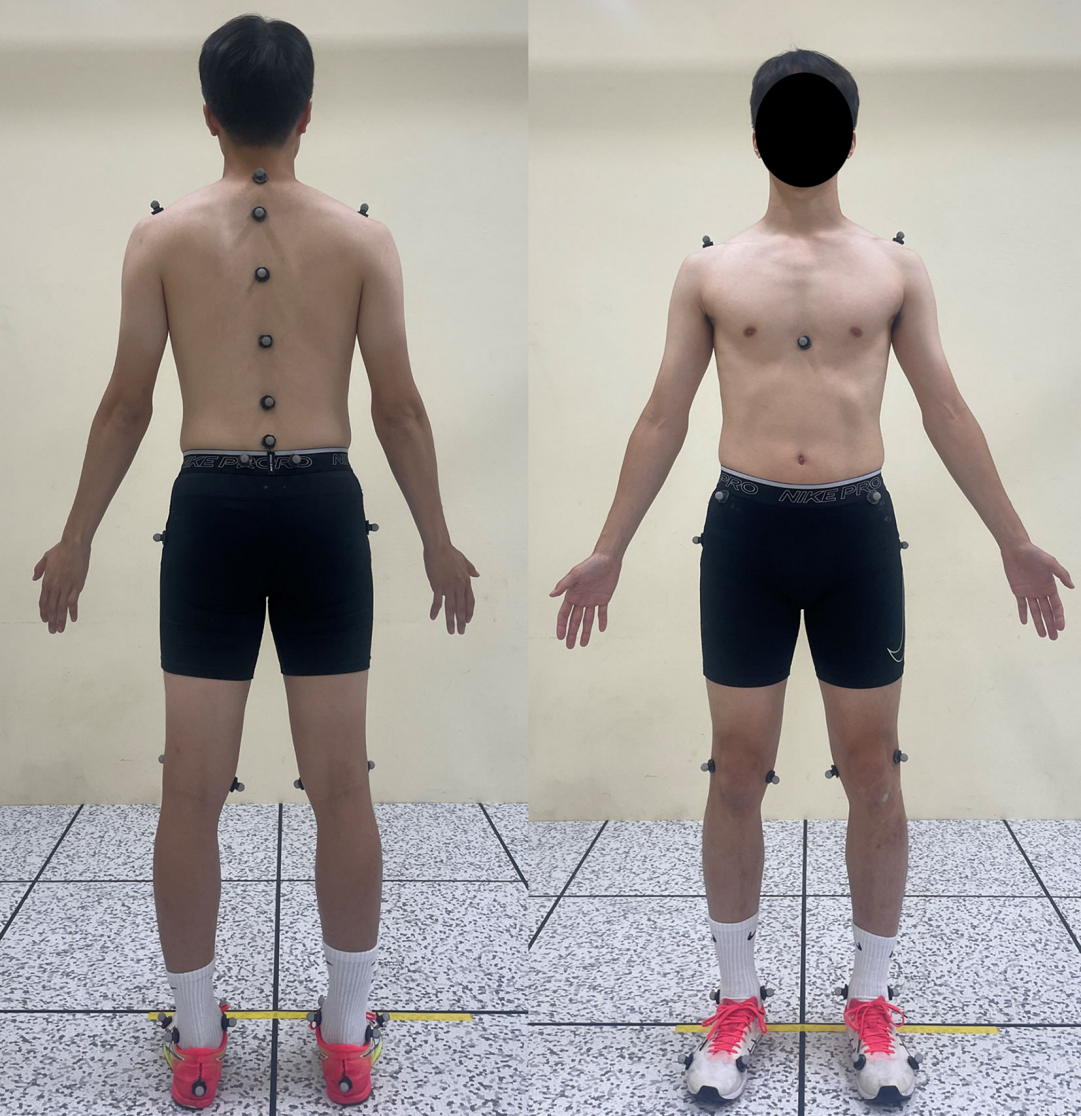
The positions of markers used in this study.

Every participant walked on an instrumented treadmill (Bertec Inc., Columbus, OH, USA) at their PWS during three trials. Meyer *et al*. suggested that 6 min are required for treadmill familiarization in a single trial [23], and other studies employed modified experimental protocols with PWS measurement and multiple trials [24,25] based on the study by Meyer *et al*. In this study, we consulted these previous studies and assumed trial-dependent familiarization time: 5 min for the first trial and 2 min for the second and third. Thus, we let the participants walk for 15 min during the first trial and 12 min during each of the second and third trials to use the data of 10 min walking for each trial. The participants rested for 10 min between trials to mitigate the potential effects of fatigue. We obtained the kinematic data using 29 reflective markers and 17 infrared cameras (OptiTrack PrimeX 13; NaturalPoint, Inc., Corvallis, OR, USA). The reflective markers were placed on the anatomical landmarks at the lower extremity and upper body trunk (figure 1). For the lower extremity, we placed the markers on the first and fifth metatarsals, hill, medial and lateral malleolus, medial and lateral epicondyle, greater trochanter and anterior–posterior iliac spine. For upper body trunk, we placed the markers on the seventh cervical vertebra (C7), the second (T2), sixth (T6) and tenth thoracic vertebra (T10), the second (L2) and fifth lumbar vertebra (L5), the sternum and both shoulders. This set of markers is a modified version of Rizzoli Markerset [26,27]; we excluded the markers placed on soft tissues from the original Rizzoli Markerset because the positions of those markers are prone to the flesh movement.

### 2.3. Data pre-processing

The position data of each marker were filtered by a low-pass Butterworth filter with a cut-off frequency of 12 Hz [28]. The velocity and acceleration time series were derived by numerically differentiating the position data. Since we discarded the data measured in adaptation at the start, we extracted the data of 10 min walking and collected 450 strides or more from each trial. The beginning of each gait cycle was identified as the moment of the heel strike of the right foot, which was estimated as the moment when the marker on the right heel was furthest away from the midpoint between the right and left posterior superior iliac spine. Consulting the previous study by Raffalt *et al*. [29], we normalized each gait cycle as 100 data points using linear interpolation. Thus, at least 45,000 data points were obtained for each trial of each participant.

### 2.4. Maximal Lyapunov exponent

Consulting previous studies that measured MLE of human walking [1–4,9–13,15,17,19,22,30–33], we chose 18 kinds of frequently used kinematic data: velocity and acceleration of T2, T10 and L5 in anteroposterior (AP), mediolateral (ML) and vertical (VT) directions. For the phase space reconstruction, we set the embedding dimension as 5 (*dE* = 5), consulting a previous study [34]. For the time delay, we used two types: the fixed delay of 10% of the gait cycle and the delay selected by the AMI algorithm. The length of the input time series varied from 10 to 450 strides with the increment of 10 strides. The MLE was calculated using Rosenstein’s algorithm [35]. Based on the recommendation of Reynard *et al*. [1], the region for the linear regression on the divergence plot was set to 0–0.5 strides. The source code used for MLE calculation is included in the electronic supplementary material.

### 2.5. Test–retest reliability

The MLEs obtained from the three trials of 15 participants were used to measure intertrial reliability. The estimated values of the ICC and their 95% confidence intervals were measured based on the single-rating, absolute-agreement, two-way mixed-effects model. The ICC criteria are as follows: poor (0–0.5), moderate (0.5–0.75), good (0.75–0.9) and excellent (0.9–1.0) [36]. We calculated ICC for each data length between 10 and 450, and then identified the minimum data length that yielded ICC over 0.75 for each type of delay.

### 2.6. Linear mixed model

Statistical analysis was performed using Jamovi (v. 2.3.28). To assess the influence of delay and data length on the ICC, a linear mixed model (LMM) was employed. The LMM was chosen for its ability to handle relationships between predictors and the response variable. Specifically, the model included delay and data length as fixed effects, with types of the time series as a random effect to account for potential variability across different types of the time series. A −log(1 − *y*) transformation was used in conjunction with a Gaussian distribution for the response variable. Marginal and conditional *R*^2^ values were calculated to quantify the variance explained by the fixed effects alone and by the entire model (fixed and random effects), respectively.

## 3. Results

**Figure 2.**
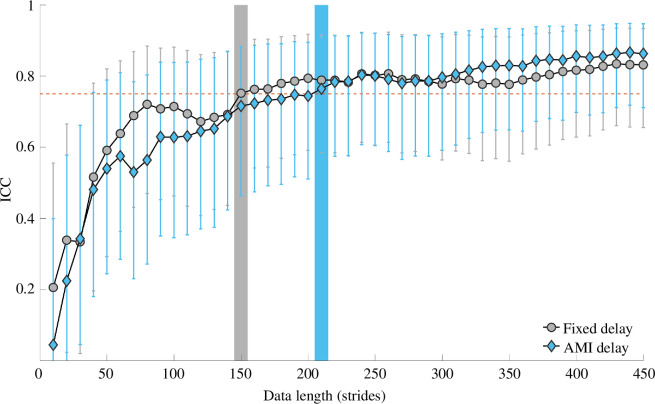
The dependence of ICC on the data length and type of delay. The effects of the data length and delay type on ICC are plotted for the velocity of the T2 marker in mediolateral direction. Grey circles and blue diamonds indicate the mean values of intertrial ICC for the fixed delay and the delay from the AMI algorithm, respectively. Grey and blue error bars indicate the 95% confidence intervals of ICC for the fixed delay and the delay from the AMI algorithm, respectively. Grey and blue vertical bands indicate the data length beyond which the ICC begins to be greater than 0.75 for the fixed delay and the delay from the AMI algorithm, respectively.

Representative ICC curves depending on the data length and time delay are shown in figure 2. The ICC value of the measured MLEs typically increases as the data length or number of used strides increases. The whole data including ICC curves for all 18 types of time series and two types of delay are available in the electronic supplementary material.

Table 1 shows the ICC value for each one-dimensional time series and each time delay when the data of 450 strides were all used. Out of 18 kinds of the one-dimensional time series, 15 of them achieved ICC over 0.75. The ICC values for T10 ML velocity and T10 VT acceleration fell within the moderate range (0.5 < ICC < 0.75), and L5 ML velocity showed poor reliability (ICC < 0.5).

**Table 1.** Intertrial ICCs of one-dimensional time series of 450 strides with two delay types. (acc, acceleration; AP, anteroposterior; ML, mediolateral; vel, velocity; VT, vertical.)

marker	data type	AMI delay (gait %)	ICC [95% CI]	fixed delay (gait %)	ICC [95% CI]
T2	AP vel	16	0.85 [0.68,0.94]	10	0.92 [0.81,0.97]
	ML vel	30	0.86 [0.71,0.95]	10	0.83 [0.66,0.93]
	VT vel	12	0.95 [0.89,0.98]	10	0.93 [0.85,0.97]
	AP acc	11	0.96 [0.91,0.99]	10	0.97 [0.92,0.99]
	ML acc	17	0.92 [0.83,0.97]	10	0.92 [0.82,0.97]
	VT acc	10	0.79 [0.58,0.92]	10	0.79 [0.58,0.92]
T10	AP vel	15	0.86 [0.71,0.95]	10	0.84 [0.67,0.94]
	ML vel	24	0.72 [0.48,0.88]	10	0.72 [0.48,0.89]
	VT vel	12	0.93 [0.85,0.97]	10	0.91 [0.80,0.96]
	AP acc	11	0.85 [0.70,0.94]	10	0.86 [0.70,0.94]
	ML acc	13	0.85 [0.69,0.94]	10	0.82 [0.63,0.93]
	VT acc	10	0.71 [0.47,0.88]	10	0.71 [0.47,0.88]
L5	AP vel	15	0.80 [0.61,0.92]	10	0.83 [0.65,0.93]
	ML vel	22	0.49 [0.18,0.76]	10	0.64 [0.37,0.85]
	VT vel	11	0.96 [0.90,0.98]	10	0.94 [0.87,0.98]
	AP acc	9	0.91 [0.80,0.96]	10	0.90 [0.78,0.96]
	ML acc	8	0.92 [0.83,0.97]	10	0.94 [0.87,0.98]
	VT acc	9	0.90 [0.78,0.96]	10	0.87 [0.73,0.95]

Table 2 shows the number of strides beyond which the ICC value becomes greater than 0.75 for each one-dimensional time series and each time delay. Among the 15 types of the one-dimensional time series, which eventually yielded ICC over 0.75 within 450 strides, the data lengths necessary for achieving ICC over 0.75 varied from 20 to 320 strides.

**Table 2.** Minimum data lengths for achieving ICC over 0.75 for each one-dimensional time series and delay type. (Note*:* the dash indicates that the ICC value stays below 0.75 for all tested data lengths. acc, acceleration; AP, anteroposterior; ML, mediolateral; vel, velocity; VT, vertical.)

marker	data type	minimum data length (strides)	
		fixed delay	AMI delay
T2	AP vel	210	320
	ML vel	150	210
	VT vel	110	90
	AP acc	30	30
	ML acc	30	40
	VT acc	290	290
T10	AP vel	90	80
	ML vel	—	—
	VT vel	80	30
	AP acc	120	130
	ML acc	250	100
	VT acc	—	—
L5	AP vel	40	80
	ML vel	—	—
	VT vel	30	30
	AP acc	40	60
	ML acc	40	40
	VT acc	20	30

The analysis using an LMM with a −log(1 − *y*) transformation revealed significant effects of data length on the ICC but no significant effect of delay or interaction between delay and data length. The model converged successfully using the ‘BOBYQA’ optimizer. The marginal *R*^2^ was 0.287, whereas the conditional *R*^2^ was 0.874, indicating that the transformation is proper for the model. Additionally, the skewness and kurtosis of residuals are, respectively, 0.198 and 2.18, justifying the use of Gaussian distribution. The effect of series length on ICC was highly significant (*F* = 83.4299, *p* < 0.001). The parameter estimates for data length show a significant increase in ICC with longer data lengths, indicating a substantial improvement in reliability as the series length increases from 10 to 450 strides. The high conditional *R*^2^ value suggests that the ICC curve is curvilinear. Conversely, the fixed effects analysis revealed that there is no effect of the types of delay on ICC; the effect of time delay on ICC was not significant (*F* = 0.0736, *p* = 0.786). The interaction between time delay and series length was not statistically significant (*F* = 0.2692, *p* = 1.000). This suggests that the relationship between series length and ICC is consistent across different delay settings. The random effect of the time series was also considered in the model, with the standard deviation of the random intercept being 0.536. This highlights considerable between-marker variability. Despite this variability, the fixed effects of series length on ICC remain consistent across different marker types.

## 4. Discussion

In multiple previous studies, the local dynamic stability of human walking has been assessed by estimating MLE [1–5,9–19,22,29–34]. However, various choices of time series of kinematic data, data length and methods for selecting the time delay have been almost arbitrarily adopted without solid justification. In this study, we demonstrate the dependence of the reliability of MLE assessment on the type of one-dimensional time series, data length and time delay and suggest some specific choices that can yield high reliability even with limited lengths of data.

In general, the reliability of estimations of indices is expected to increase as the amount of the data increases. Our result is consistent with this prediction. As illustrated in figure 2, ICC generally increases as the length of used data increases. We deliberately chose 18 kinds of time series of kinematic data that have been frequently used for MLE estimations [1–4,9–13,15,17,19,22,30–33]. For the majority of the chosen time series, the ICC of MLE estimations culminated in high to excellent levels (ICC > 0.75) when 450 strides, which typically required approximately 10 min walking at the participants’ preferred speed, were employed. There were three exceptions: the time series of T10 ML velocity, T10 VT acceleration and L5 ML velocity consistently resulted in moderate (0.5 < ICC < 0.75) or poor (ICC < 0.5) ICCs regardless of delay type even when the entire 450 strides were used (table 1). We also found that the minimum data length required for good test–retest reliability heavily depends on the choice of one-dimensional time series. Even among the 15 kinds of time series that yield ICC above 0.75 within 450 strides, the minimum data length beyond which ICC becomes greater than 0.75 ranges from 20 to 320 strides (table 2).

Our results enable us to identify a group of one-dimensional time series that allows efficient and reliable estimations of the local dynamic stability of human walking. These high-performing time series demonstrate two key strengths: they achieve good test–retest reliability (ICC > 0.75) with relatively short data lengths of 60 strides or less and further exhibit excellent reliability (ICC > 0.9) when the data length approaches 450 strides or 10 min walking. This group comprises T2 AP acceleration, T2 ML acceleration, L5 VT velocity, L5 AP acceleration and L5 ML acceleration. The previous study by Reynard *et al*. [1], which successfully differentiated healthy controls from patients with neurological disorders only with 35 steps using lumbar acceleration data, further supports the suitability of our results.

Our findings are particularly important considering the trade-off between shortening the experimental session and achieving high reliability. A part of our results confirms the benefits of longer data for improved test–retest reliability in most time series, but this advantage comes at the cost of long experiment duration, which gait-impaired individuals may not be able to endure. For example, it may be implausible that the neurologically impaired population or the elderly walk for 15 min, enabling the acquisition of sufficient data for reliable estimations of MLE. Even for studies in which only unimpaired individuals participate, we frequently need to minimize the experiment duration to avoid any possible artefact owing to adaptation, learning or fatigue. The results summarized in tables 1 and 2 can contribute to addressing this critical trade-off; we provide a comprehensive map of reliability values across various time series and data lengths. This achievement allows researchers to tailor their experimental protocols so that they estimate MLE with desired reliability levels under the time constraints of practically acceptable experimental sessions.

If we intend to estimate MLE using infinite noise-free data from a deterministic system, the time delay can be chosen arbitrarily [7]. However, any dataset from actual measurement, including the dataset from human locomotion, inevitably has noise and limited length. In such a case, excessively small or large time delay can significantly distort the topological features of the original system and the resultant assessment of MLE [8,37]. The AMI algorithm, utilizing Shannon’s information theory [38], offers a data-driven approach to resolving this challenge [7]. Although multiple previous gait studies have employed this AMI algorithm, its influence on the test–retest reliability of MLE estimations has remained untested. Our findings show that the time delay chosen by the AMI algorithm does not outperform the fixed delay in yielding high reliability of estimations of MLE of human walking.

A systematic review of studies that have involved estimations of MLE of human walking reveals the wide variety of the estimated MLE values [20]. Even if MLE was estimated from the time series of the kinematics of a single marker attached to a single participant, the MLE value varied significantly depending on the degree of freedom of the marker data. For example, there can be a significant difference between the MLE values estimated from the time series of L5 AP acceleration and those estimated from the time series of L5 ML acceleration [1]. This sensitive dependence of MLE values on the choice of the kinematic data within a participant and within a trial devalues the attempts to assess the validity of MLE estimations by cross-checking the MLE values estimated from a single participant. The clear dependence of MLE values on the estimation method rather clarifies the need for controlling the estimation method and using the MLE values only for comparison, e.g. between with and without any intervention or impairment. Based on the known sensitive dependence of MLE estimations on the choice of the kinematic data, we confined the scope of this study to the test–retest reliability of MLE estimations. The validity of the MLE estimation, which is hardly measurable unless we can access the true MLE value of individual human walking, was not included in the scope of this study.

Another limitation of this initial study is that we focus only on a homogeneous group of young and healthy males; this reduces the generalizability of our results to broader populations. Notably, the influence of age, gender and other demographic factors on the reliability of MLE estimations remains unexplored. However, establishing the criteria for measuring MLE of the young and healthy group alone is practically important. When designing experiments to assess the effects of various conditions on MLE, it is typically essential to measure the MLE of a young and healthy control group for comparison with the experimental group. Therefore, ensuring the reliability of MLE in these control groups is crucial by itself. Related future studies might include different groups, such as patients and the elderly. However, such studies need to address the challenges owing to insufficient data length; individuals with gait disabilities may not be able to complete sufficiently long walking sessions.

In addition, to achieve the original aim of assessing the reliability of relatively frequently accepted methods for estimating MLE, this study reported the estimation of MLE based on trunk kinematics only. However, depending on the experimental set-up, kinematic data of the lower extremities may be more easily accessible. How the reliability of MLE depends on the kinematic variable, data length and time delay when MLE is estimated by the joint angles of the lower extremities needs to be addressed in future studies.

It is important to note that MLE estimation depends on numerous factors. We focused on data type, data length and time delay in this study, but other factors, such as embedding dimensions, regression regions, normalization methods and experimental conditions, can also affect MLE values. Although analysing the effects of all parameters would be beneficial, our initial focus was on the three factors which particularly seem to be arbitrarily chosen in previous studies. The effect of other factors on MLE estimation needs be evaluated in future work.

Various techniques in applied mathematics have been used to assess the dynamics of human movement, and the estimation of MLE is one representative example. However, it is challenging to confirm whether the commonly used methods yield reliable outcomes. This study investigated the effect of choice of one-dimensional time series, time delay and data length on the test–retest reliability of MLE estimations in gait analysis, to our knowledge, for the first time. Our findings reveal that some specific choices of time series can yield excellent reliability even with relatively short data lengths. Interestingly, no significant difference in the reliability emerged between estimates obtained using the delay from the AMI algorithm and fixed delay. These findings will contribute to establishing guidance on designing reliable and efficient gait analysis experiment protocols and data analyses and enhancing the clinical applicability of MLE.

## Data Availability

Data and code associated with this work are available in the electronic supplementary material [39].
